# A neural circuit associated with anxiety‐like behaviors induced by chronic inflammatory pain and the anxiolytic effects of electroacupuncture

**DOI:** 10.1111/cns.14520

**Published:** 2023-11-29

**Authors:** Zemin Wu, Zui Shen, Yingling Xu, Shaozong Chen, Siqi Xiao, Jiayu Ye, Haiyan Zhang, Xinyi Ma, Yichen Zhu, Xixiao Zhu, Yongliang Jiang, Junfan Fang, Boyi Liu, Xiaofen He, Shuzhong Gao, Xiaomei Shao, Jinggen Liu, Jianqiao Fang

**Affiliations:** ^1^ Key Laboratory of Acupuncture and Neurology of Zhejiang Province, Department of Neurobiology and Acupuncture Research The Third Affiliated Hospital of Zhejiang Chinese Medical University Hangzhou China; ^2^ Department of Acupuncture and Moxibustion the First Affiliated Hospital of Zhejiang Chinese Medical University Hangzhou China; ^3^ Liangzhu Laboratory Zhejiang University Medical Center Hangzhou China; ^4^ Institution of Acupuncture and Moxibustion, Shandong University of Traditional Chinese Medicine Jinan China; ^5^ National Key Laboratory of Drug Research Shanghai Institute of Materia Medica, Chinese Academy of Sciences Shanghai China; ^6^ Key Laboratory of Neuropharmacology and Translational Medicine of Zhejiang Province, School of Pharmaceutical Sciences Zhejiang Chinese Medical University Hangzhou China

**Keywords:** anxiety‐like behavior, chronic inflammatory pain, dorsal raphe nucleus, electroacupuncture, rostral anterior cingulate cortex, serotonergic neurons

## Abstract

**Aims:**

Negative emotions induced by chronic pain are a serious clinical problem. Electroacupuncture (EA) is a clinically proven safe and effective method to manage pain‐related negative emotions. However, the circuit mechanisms underlying the effect of EA treatment on negative emotions remain unclear.

**Methods:**

Plantar injection of complete Freund's adjuvant (CFA) was performed to establish a rat model of chronic inflammatory pain‐induced anxiety‐like behaviors. Adeno‐associated virus (AAV) tracing was used to identify excitatory synaptic transmission from the rostral anterior cingulate cortex (rACC) to the dorsal raphe nucleus (DRN). Employing chemogenetic approaches, we examined the role of the rACC‐DRN circuit in chronic pain‐induced anxiety‐like behaviors and investigated whether EA could reverse chronic pain‐induced dysfunctions of the rACC‐DRN circuit and anxiety‐like behaviors.

**Results:**

We found that chemogenetic activation of the rACC‐DRN circuit alleviated CFA‐induced anxiety‐like behaviors, while chemogenetic inhibition of the rACC‐DRN circuit resulted in short‐term CFA‐induced anxiety‐like behaviors. Further research revealed that the development of CFA‐induced anxiety‐like behaviors was attributed to the dysfunction of rACC CaMKII neurons projecting to DRN serotonergic neurons (rACC^CaMKII^‐DRN^5‐HT^ neurons) but not rACC CaMKII neurons projecting to DRN GABAergic neurons (rACC^CaMKII^‐DRN^GABA^ neurons). This is supported by the findings that chemogenetic activation of the rACC^CaMKII^‐DRN^5‐HT^ circuit alleviates anxiety‐like behaviors in rats with chronic pain, whereas neither chemogenetic inhibition nor chemogenetic activation of the rACC^CaMKII^‐DRN^GABA^ circuit altered CFA chronic pain‐evoked anxiety‐like behaviors in rats. More importantly, we found that EA could reverse chronic pain‐induced changes in the activity of rACC CaMKII neurons and DRN 5‐HTergic neurons and that chemogenetic inhibition of the rACC^CaMKII^‐DRN^5‐HT^ circuit blocked the therapeutic effects of EA on chronic pain‐induced anxiety‐like behaviors.

**Conclusions:**

Our data suggest that the reversal of rACC^CaMKII^‐DRN^5‐HT^ circuit dysfunction may be a mechanism underlying the therapeutic effect of EA on chronic pain‐induced anxiety‐like behaviors.

## Graphical abstract

Chronic inflammatory pain induces anxiety‐like behaviors, and electroacupuncture ameliorates negative emotions by intervening rACC^CaMKII^‐DRN^5‐HT^ neural circuit but not the rACC^CaMKII^‐DRN^GABA^ circuit.

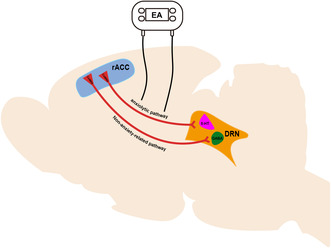

## 1 INTRODUCTION

Chronic pain and anxiety are highly comorbid.^1^, ^2^ Epidemiological studies show that 44%–70% of chronic pain patients have long‐term anxiety symptoms.^3^, ^4^, ^5^ Long‐term anxiety can exacerbate pain, which in turn causes more serious anxiodepressive disorders.^2^, ^6^, ^7^ The prevention and treatment of chronic pain‐induced anxiety is an important clinical issue that urgently needs to be solved. Electroacupuncture (EA) is a nonpharmacological external treatment method and has been widely used for chronic pain, emotional disorders, and the comorbidity of pain and anxiety.^8^, ^9^, ^10^, ^11^ Distinct from drugs that are currently recommended for the treatment of anxiety associated with chronic pain, treatment with EA has only minor side effects.^12^ Despite the increasing use of EA in treating anxiety associated with chronic pain, the mechanisms by which EA contributes to ameliorating anxiety associated with chronic pain are still unknown.

The comorbidity of chronic pain and anxiety may be the consequence of specific dysfunctions in the functional and structural connectivity of the neural circuits that are integral in controlling sensory, emotional, and cognitive functions. The anterior cingulate cortex (ACC) may be a strong candidate for the interaction of pain and anxiety because it has extensive afferent and efferent connections with both cortical and subcortical structures.^13^ The involvement of the ACC in pain and anxiety has been reported in human and animal studies.^14^, ^15^, ^16^, ^17^ Our previous study also supports the involvement of the rostral ACC (rACC) in anxiety‐like behaviors associated with chronic pain in rats.^18^ Although substantial evidence supports that the rACC plays an important role in pain‐related mood disorders, the circuit mechanisms underlying rACC‐mediated development of the comorbidity of chronic pain and mood disorders remain unclear.

The serotonergic system has been the focus of preclinical and clinical studies on the relationship between pain and mood disorders. A decrease in 5‐HT levels in the prefrontal cortex in rats has been shown in meningeal nociception (resembling chronic migraine in humans) induced by infusing inflammatory agents into the dura mater of rats.^19^ On the other hand, the selective serotonin reuptake inhibitor fluoxetine blocks the anxiodepressive‐like consequences induced by sciatic nerve injury.^20^ These findings highlight the role of serotonin signaling in modulating the comorbidity of chronic pain and anxiodepressive disorders. Given that the dorsal raphe nucleus (DRN) is rich in serotonergic neurons (5‐HTergic neurons) and is well known to be one of the most important regions for the regulation of anxiodepressive disorders, it is highly possible that the DRN may be a key subcortical structure that may integrate anxiety signaling either directly or indirectly through projections from the rACC. Our previous studies have shown that EA can improve pain‐related anxiety or depression by intervening in the rACC and DRN, respectively.^21^, ^22^ Given the projection relationship between the rACC and DRN, we hypothesized that EA may improve pain‐induced anxiety‐like behaviors through the rACC‐DRN circuit. The present study, therefore, was undertaken to test the hypothesis that dysfunction of the rACC‐DRN circuit might play a special role in chronic pain‐related anxiety and to investigate the mechanism by which EA ameliorates chronic pain‐induced anxiety‐like behaviors. By employing a combination of anatomical tracing and chemogenetic manipulations, we showed that the rACC was reciprocally connected with the DRN. Chemogenetic activation and inhibition of the rACC‐DRN circuit inversely modulated chronic pain‐induced anxiety‐like behaviors. EA can ameliorate anxiety‐like behaviors associated with chronic pain by restoring rACC‐DRN circuit functions.

## 2 MATERIALS AND METHODS

### 2.1 Animals

Male adult Sprague–Dawley (SD) rats weighing 250–300 g were obtained from the Experimental Animal Center of Zhejiang Chinese Medical University. The animals were housed in groups of 3–4 in plastic cages with soft bedding at the University Animal Care Facility and were subjected to an artificial 12/12 h light–dark cycle (lights on at 8 a.m.). Animals received food and water ad libitum, and the temperature was constant, between 23 and 25°C, with a relative humidity of 40%–70%. Before experimental manipulations, rats were given 1 week to adjust to their surroundings. All procedures were carried out following the guidelines from the International Association for the Study of Pain and the Institutional Animal Ethical Committee (IAEC).

### 2.2 Rat model of chronic pain induced anxiety‐like behavior

Chronic pain was induced by injecting 100 μL of complete Freund's adjuvant (CFA, Sigma–Aldrich, USA), which was emulsified in sterile 0.9% saline at a ratio of 1:1 into the left hind paw of each rat. The same amount of sterile 0.9% saline was injected into the control rats in the same way.

The successful modeling was determined through the observation of anxiety‐like behavior in the animal without any effect on motor function.^1^, ^23^

### 2.3 Stereotaxic surgery and drugs

Quinolinic acid (QA, Sigma–Aldrich, USA) was dissolved in sterile 0.9% saline and adjusted to a pH of 7.2–7.4 by using 0.1 M NaOH at a final concentration of 200 nmol/μL. 5,7‐Dihydroxtryptamine (5,7‐DHT, Sigma–Aldrich, USA) was dissolved in sterile 0.9% saline with 0.05% ascorbic acid, and the final concentration was 10 μg/μL.

The rACC was bilaterally lesioned by injection of 400 nL QA solution at a rate of 50 nL/min on both sides. The coordinates of all solutions injected in the rACC were as follows: AP: 3.00 mm, ML: 0.8 mm, and DV: 1.4 mm. The DRN was lesioned by injection of 500 nL 5,7‐DHT solution at a rate of 50 nL/min. The coordinates of all solutions injected in the DRN were as follows: AP: −7.80 mm, ML: 2.28 mm at an angle of 20 degrees for targeting, and DV: 6.17 mm. For surgery, rats were anesthetized with sodium pentobarbital (60 mg/kg) and then securely placed into a stereotaxic frame (RWD Life Science, China) and onto a heating pad set at 37°C. A small cranial cavity was made using a dental drill (RWD Life Science, China). QA was injected into the rACC through a glass microelectrode connected to a microinjection syringe (Nanofil, WPI, USA) and its controller (Micro4, WPI, USA). 5,7‐DHT was injected into the DRN via a microinjection syringe with a 33‐gauge needle (NF33BV‐2, WPI, USA). The syringe remained on the site for 10 min after injection to avoid drug overflow. Rats were placed in a 37°C environment until they awakened after the operation. Surgeries were performed 1 week before the models were established to ensure that rats would recover from the operation. When all experiments were finished, brain slices were taken and analyzed to confirm the injection location.

### 2.4 Virus infusion

Virus infusions were performed by the same method as in the drug injection described above. Four hundred nanoliters of virus was injected into the rACC. The glass microelectrode was left in place for 10 min after injection to prevent leakage of the virus. Three hundred nanoliters of each virus was injected into the DRN. For a mixture of two viruses, the injection volume was 600 nL split into three injections of 200 nL each, with a 20 min interval between injections to prevent virus leakage.

For anterograde tracing, AAV2/9‐CaMKII‐EGFP (virus titers: 5.34E+12 vg/mL) was delivered into the right rACC of rats to label the somas of CaMKII projection neurons. Rats were then kept in cages for 6 weeks to ensure adequate virus expression.

For retrograde tracing, AAV2/R‐CaMKII‐EGFP (virus titers: 5.24E+12 vg/mL) was delivered into the DRN of rats to label the termini of the CaMKII neurons. Rats were then kept in cages for 6 weeks to ensure adequate virus expression.

To regulate the DRN‐projecting rACC CaMKII neurons by using designer receptors exclusively activated by designer drugs (DREADDs), the Cre‐dependent virus (AAV2/9‐CaMKII‐DIO‐hM3D‐mCherry, virus titers: 5.32E+12 vg/mL; AAV2/9‐CaMKII‐ DIO‐hM4D‐mCherry, virus titers: 5.22E+12 vg/mL) (depending on activation or inhibition) was injected into the rACC of rats, and AAV2/R‐CaMKII‐Cre (virus titers: 6.24E+12 vg/mL) was injected into the DRN to specifically retrogradely infect CaMKII neurons projecting from the rACC. Rats that received AAV perfusion carrying hM4D receptors received bilateral injections into the rACC, and those that received hM3D receptors received an injection into the right rACC.

To specifically label the DRN serotonergic neurons that receive rACC inputs, a 1.8‐kb fragment containing a part of exon 1 and the 5′ flanking region of the mouse Pet1 gene was intercepted as the Pet1 promoter according to the methods of Hasegawa et al.^24^ The AAV‐Pet1‐cre virus used in this study is based on the above method. AAV2/1‐hSyn‐Flp (virus titers: 1.03E+13 vg/mL) was injected into the right rACC of rats to allow anterograde spread into downstream postsynaptic neurons and Cre expression. Simultaneously, a mixed solution of Cre and Flp‐dependent AAV (AAV2/9‐hSyn‐Con/Fon‐hM3D‐EGFP, virus titers: 5.03E+12 vg/mL) and AAV2/9‐Pet1‐Cre (virus titers: 1.63E+13 vg/mL, Taitool, China) was injected into the DRN.

To regulate DRN GABAergic neurons, AAV2/9‐VGAT1‐hM3D‐mCherry or AAV2/9‐VGAT1‐hM4D‐mCherry was injected into the DRN to specifically infect local GABAergic neurons.

To inhibit or activate the DRN GABAergic neurons that receive rACC inputs, AAV2/1‐hSyn‐Cre (virus titers: 1.00E+13 vg/mL) was injected bilaterally into the rACC of rats, and AAV2/9‐VGAT1‐DIO‐hM4D‐mCherry (virus titers: 2.86E+12 vg/mL) or AAV2/9‐VGAT1‐DIO‐hM3D‐mCherry (virus titers: 2.00E+12 vg/mL) was injected into the DRN to specifically label the DRN GABAergic neurons that receive CaMKII neurons inputs from the rACC.

Unless otherwise stated, all viruses were packaged by BrainVTA (China).

### 2.5 Nociceptive behavioral testing

A dynamic plantar aesthesiometer (Ugo Basile, Italy) was used to measure paw withdrawal thresholds (PWTs). Rats were placed individually in transparent plastic chambers positioned on a wire mesh grid. A paw‐flick response was elicited by applying an increasing vertical force (increased steadily from 0 to 50 g over 20 s) using a stainless steel probe (a straight 0.5 mm diameter shaft) placed underneath the mesh floor and positioned at the middle of the plantar surface of the left hind paw. According to our previous study, PWTs were determined as the mean of four subsequent measurements, except for the first, at intervals of 1 min.^21^ The PWT testing procedure is shown in the results.

### 2.6 EA treatment

In our previous study, we found that bilateral stimulation of the “Zusanli” (ST36) and “Sanyinjiao” (SP06) acupoints for 1 h (the initial strength is 0.5 mA, increasing by 0.5 mA every 20 min) at a frequency of 100 Hz could relieve anxiety‐like behaviors induced by chronic inflammatory pain.^25^ Therefore, we chose the same acupoints and parameters to treat the rats in the EA group. The acupoints were needled with stainless steel acupuncture needles (0.25 mm in diameter × 13 mm in length) and electrically stimulated with a Hans Acupoint Nerve Stimulator (HANS 200A, China). During EA treatment, a special cotton retainer was used to immobilize the rats to prevent struggling. During the whole procedure, all rats appeared to remain in relatively comfortable states without any struggling or screaming. Rats in the other groups received the same restraint treatment to counterbalance the effects of restraint. EA treatment was performed before each behavioral test. After EA was completed, the rats were placed in a rat cage to allow them to calm down, and behavioral experiments were performed approximately 10 min later. Each rat was placed in a separate cage with clean, soft bedding at the end of the test to avoid disturbing rats in the same cage that were not performing the test.

The rats were sacrificed 90 min after the end of the behavioral tests for immunofluorescence staining.

### 2.7 Assessment of anxiety‐like behaviors

Open field (OF) test. The OF apparatus was a brown wooden box that was 100 × 100 × 60 cm^3^ in size. The middle square area (50 × 50 cm^2^) was defined as the center area, and the remaining area was defined as the peripheral area. Rats were placed in the center of the apparatus and were allowed to explore freely for 30 s. Then, the movement trajectories of the rats for the next 5 min were recorded using Anymaze software (Stoelting, USA). The amount of time rats spent in the central area was recorded as an indicator of the degree of anxiety. The box was wiped clean with 75% alcohol after each test session to prevent the odor of the rat that was previously in the apparatus from affecting the behavior of the next rat.

EPM test. The EPM apparatus consisted of two opposing open arms (10 × 100 × 1 cm^3^) and two opposing closed arms (10 × 100 × 40 cm^3^). The open arms and the closed arms were connected by a square (10 × 10 cm^2^) called the center area. The apparatus was 60 cm above the floor. Rats were placed in the center area with their head facing an open arm. Rats were allowed 30 s to adapt to the environment. The time rats spent in the open arms over the next 5 min was recorded using Anymaze software as an indicator of anxiety. The maze was cleaned using 75% alcohol after each test session.

Elevated zero maze (EZM) test. The EZM was a ring‐shaped apparatus consisting of a 20 cm wide circular platform. The platform was divided into four quadrants of equal length with two opposing open arms and two closed arms (surrounded by 20 cm walls on both inner and outer sides). The apparatus was 60 cm above the floor. For EZM testing, rats were placed in the junction of an open arm and an adjacent closed arm with its head facing the open arm. They were given 30 s to become accustomed to the maze, and their movement trajectories in the open arms were recorded for the next 5 min. The maze was cleaned using 75% alcohol after each test session.

All rats were sacrificed 90 min after behavioral tests for immunofluorescence staining.

### 2.8 Chemogenetic manipulation

To fully express the virus in the nucleus, we performed manipulation 6 or 7 weeks after virus injection. Clozapine N‐oxide (2 mg/kg) (CNO, BrainVTA, China) was injected intraperitoneally 30 min before each behavioral test for neuronal regulation. For rats in the EA group, CNO was injected i.p. 30 min before EA treatment.

### 2.9 Immunofluorescence and imaging

Generally, rats were anesthetized with pentobarbital sodium and then transcardially perfused successively with precooled phosphate‐buffered solution (PBS) (0.01 M) and phosphate buffer (0.01 M) containing 4% paraformaldehyde (pH = 7.3–7.4). The brains were immersed in paraformaldehyde solution overnight and then dehydrated in a gradient of 15% and 30% sucrose solutions. Finally, coronal frozen sections 30 μm thick were prepared by using a cryostat (NX50, Thermo Fisher, USA) for tracing the EGFP signal or immunofluorescence staining.

For brains infused with virus‐carrying DREADDs, sections were placed at 37°C for 1 h after removal from the refrigerator. After washing 5 times in PBST (0.01 M PBS + 0.05% Tween‐20), sections were enclosed with blocking buffer (0.3% Triton X‐100 with 5% donkey serum in PBST) for 1 h at 37°C and then incubated with primary antibodies diluted with blocking solution, including anti‐c‐Fos (1:500, rabbit, Abcam, USA), anti‐c‐Fos (1:500, mouse, Abcam, USA), anti‐CaMKII (1:200, mouse, Abcam, USA), anti‐5‐HT (1:500, goat, Abcam, USA), anti‐GFP (1:500, chicken Abcam, USA) and anti‐GABA (1:500, rabbit, Sigma–Aldrich, USA), at 4°C for 24–48 h. Subsequently, the sections were rewarmed at 37°C for 1 h, washed 5 times, and finally incubated with secondary antibodies, including donkey anti‐rabbit IgG conjugated with DyLight 488 (1:500, Jackson ImmunoResearch, USA), donkey anti‐mouse IgG conjugated with DyLight 488 (1:500, Jackson ImmunoResearch, USA), donkey anti‐mouse IgG conjugated with DyLight 647 (1:500, Jackson ImmunoResearch, USA), donkey anti‐goat IgG conjugated with DyLight 405 (1:500, Jackson ImmunoResearch, USA), donkey anti‐goat IgG conjugated with DyLight 647 (1:500, Jackson ImmunoResearch, USA), donkey anti‐rabbit IgG conjugated with DyLight 647 (1:500, Jackson ImmunoResearch, USA) and donkey anti‐chicken IgY H&L (FITC) (1:500, Abcam, USA), for 1 h at 37°C. After washing 5 times, sections were incubated with Fluoroshield mounting medium with DAPI (Abcam, USA) and cover‐slipped. The slices were scanned and imaged using a virtual slide microscope (VS‐120, Olympus, Japan) to visualize the fluorescence signals. For the quantification of positive neurons, four or five consecutive brain slices (30 μm) per brain were randomly selected.

### 2.10 Statistical analysis

Analyses were performed, and graphs were generated using GraphPad Prism 6 (GraphPad, USA). All data are presented as the means ± standard errors of the means (SEM). The Shapiro–Wilk normality test was performed to check the normal distribution of the data. The PWTs between different groups were tested with two‐way repeated‐measures ANOVA with Bonferroni post hoc correction. One‐way ANOVA with Bonferroni post hoc correction was used for multiple comparisons. Two‐tailed unpaired Student's *t* test was used to compare two independent samples. Mann–Whitney *U* test and Kruskal–Wallis tests were used if the data did not meet the assumptions of the parametric test. Significance was indicated by *p* values <0.05.

## 3 RESULTS

### 3.1 Anxiety‐like behaviors induced by chronic inflammatory pain associated with plantar injection of complete Freund's adjuvant (CFA) were abolished by lesions of either the rACC or DRN

**FIGURE 1:**
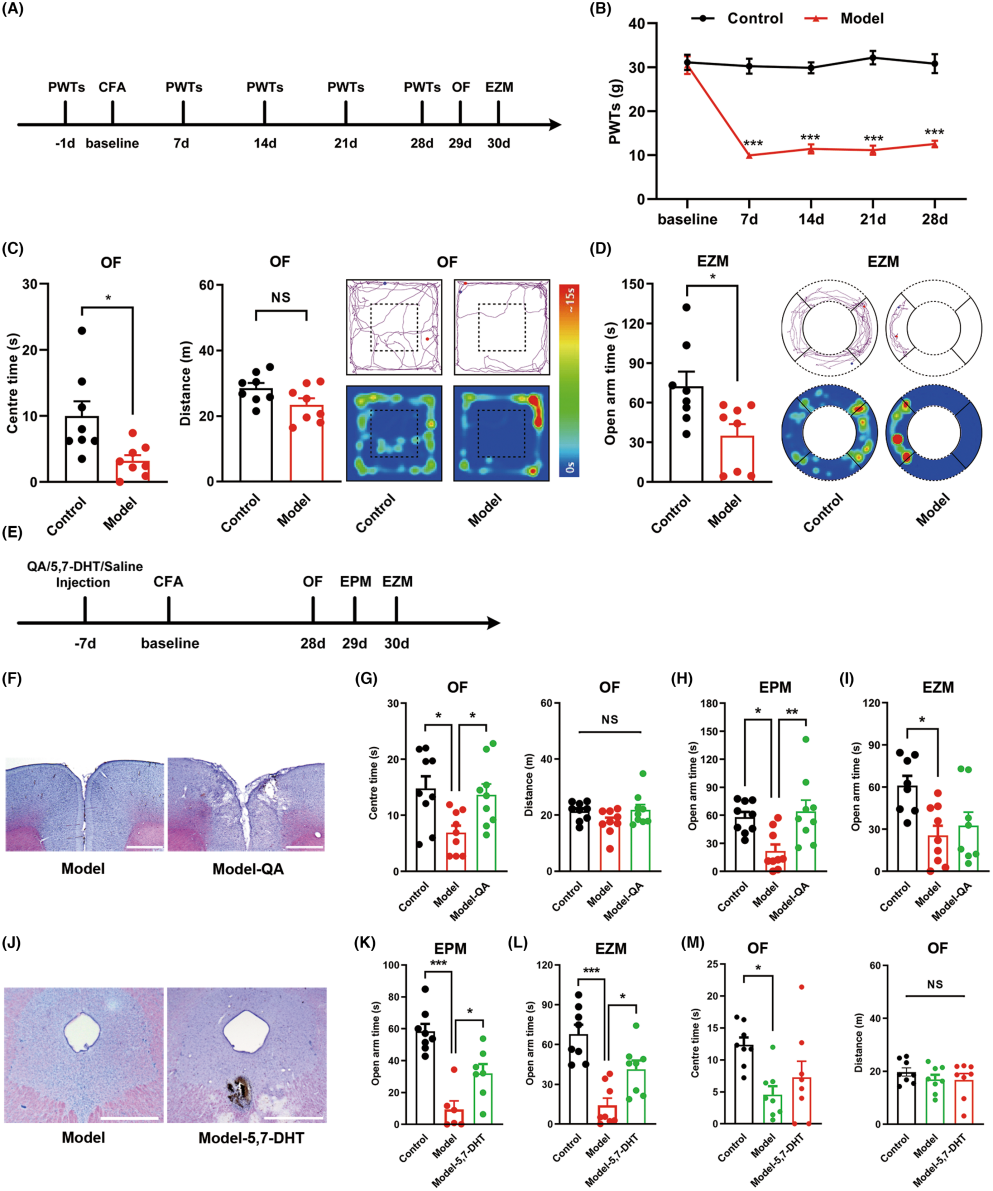
Anxiety‐like behaviors induced by chronic inflammatory pain associated with plantar injection of complete Freund's adjuvant (CFA) are abolished by lesions of either the rACC or DRN. (A) Timeline of the experimental procedure. (B) Time course of CFA‐induced changes in PWTs (*n* = 8; ***p < 0.001). (C) Time rats spent in the center area (left), total distance rats traveled (middle), and representative trajectory map and activity heatmap (right) of the OF test (*n* = 8; *p < 0.05). (D) Time rats spent in the open arms (left) and representative trajectory map and activity heatmap (right) of the EZM test (*n* = 8; *p < 0.05). (E) Timeline of the experimental procedure. (F) Photomicrographs of Nissl staining of QA‐induced rACC lesions. Scale bar: 1 mm. (G) Time rats spent in the center area (left) and total distance rats traveled (right) in the OF test (*n* = 9; *p < 0.05). (H) Time rats spent in the open arms in the EPM test (*n* = 9; *p < 0.05, **p < 0.01). (I) Time rats spent in the open arms in the EZM test (*n* = 8–9; *p < 0.05). (J) Photomicrographs of Nissl staining of 5,7‐DHT‐induced DRN lesions. Scale bar: 1 mm. (K) Time rats spent in the open arms in the EPM test (*n* = 6–8; *p < 0.05, ***p < 0.001). (L) Time rats spent in the open arms in the EZM test (*n* = 8; *p < 0.05, ***p < 0.001). (M) Time rats spent in the center area (left) and total distance rats traveled (right) in the OF test (*n* = 8; *p < 0.05). Data are represented as the mean ± SEM. Significance was assessed by two‐way repeated‐measures ANOVA with Bonferroni post hoc correction between groups (B), by two‐tailed unpaired Student's *t*‐test (C), Mann–Whitney *U* test (D), and by one‐way ANOVA with Bonferroni post hoc correction (G–I, K–M). NS: no significance.

Complete Freund's adjuvant is a common inflammatory mediator utilized to formulate pain models.^26^, ^27^ We injected 100 μL of emulsified CFA into the left hind paw to prepare a rat model of chronic inflammatory pain. The animals in the control group received the same volume of sterile 0.9% saline alone. We tested the PWTs of the rats every 7 days. Behavioral tests, including the OF test and the EZM test, were performed 4 weeks after CFA injection to investigate whether anxiety‐like behaviors occurred (Figure 1A). From the above experiments, we found that continuous nociceptive hyperalgesia was caused by plantar injections of CFA (Figure 1B), and subsequent anxiety‐like behavioral tests showed that the model rats exhibited reduced exploration in the central area of the OF and the open arms of the EZM compared with control animals (Figure 1C,D). In addition, we assayed locomotor activity and did not find any change in locomotion as measured by the total distance rats traveled (Figure 1C). These data demonstrate that CFA‐induced chronic inflammatory pain evoked persistent nociceptive hyperalgesia and anxiety‐like behaviors in rats, consistent with our previous study.^28^

The rACC and DRN were reported to be involved in negative emotions. To explore the effect of the rACC or DRN on chronic inflammatory pain‐induced anxiety‐like behavior, we injected neurotoxic drugs into the rACC or DRN to damage the nucleus. Under model conditions, rats exhibited reduced anxiety‐like behaviors following QA injection into the rACC (Figure 1E–I). Similarly, the destruction of the DRN by injection of 5,7‐dihydroxytryptamine (5,7‐DHT) in rats also reduced anxiety‐like behaviors in model rats (Figure 1E,J–M). These results provide strong evidence of the involvement of the rACC and DRN in the regulation of anxiety‐like behaviors induced by chronic inflammatory pain.

### 3.2 Chemogenetic activation or inhibition of DRN‐projecting rACC CaMKII neurons suppressed or promoted CFA‐induced anxiety‐like behaviors

**FIGURE 2:**
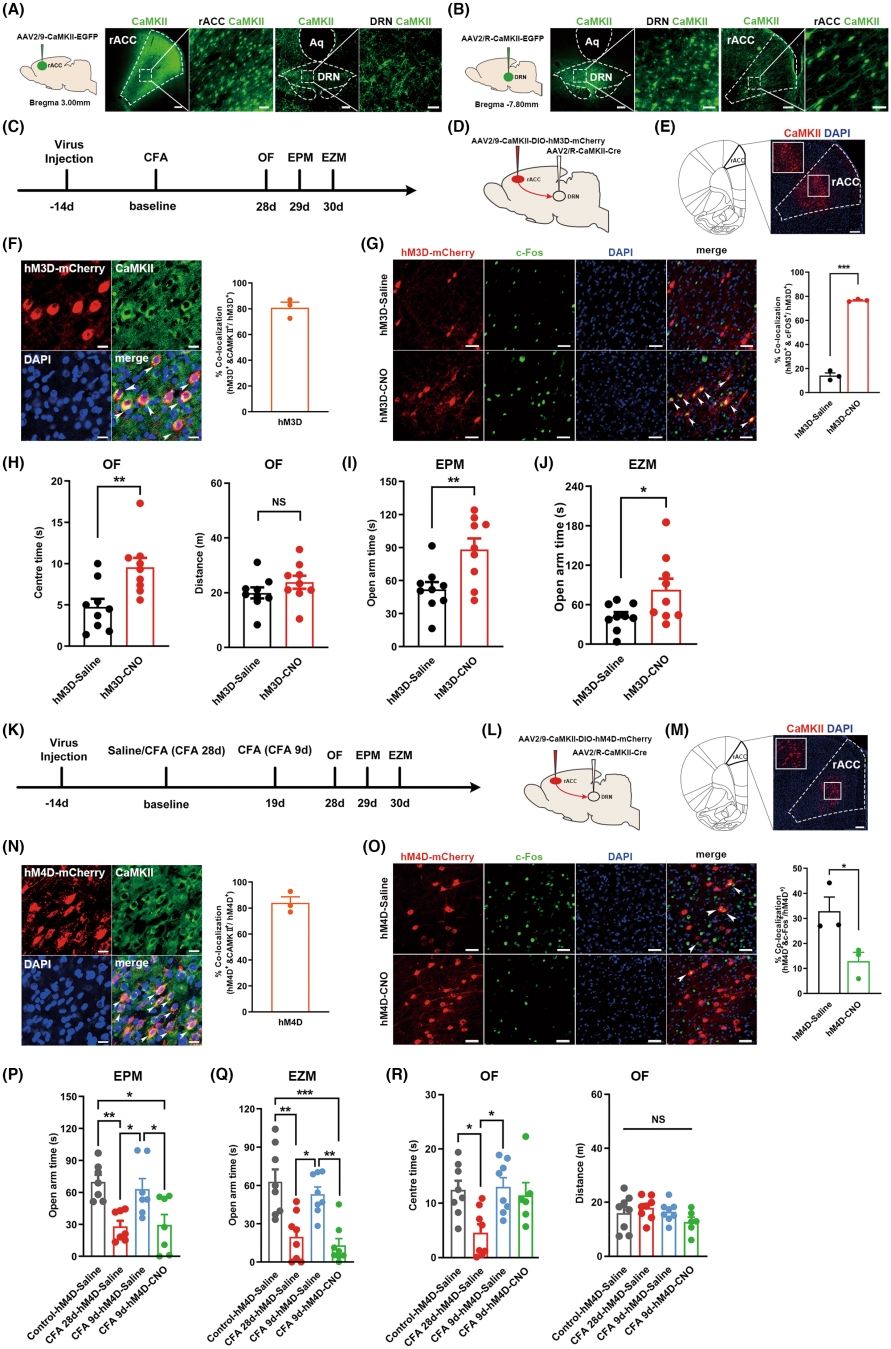
Chemogenetic activation or inhibition of DRN‐projecting rACC CaMKII neurons suppressed or promoted CFA‐induced anxiety‐like behaviors. (A) Schematic of anterograde AAV injection into the rACC (left) and representative photographs after virus injection (right). Scale bar: 200 μm for CaMKII fluorescence images, 50 μm for rACC CaMKII and DRN CaMKII fluorescence images. (B) Schematic of retrograde AAV injection into the DRN (left) and representative photographs after virus injection (right). Scale bar: 200 μm for CaMKII fluorescence images, 50 μm for DRN CaMKII and rACC CaMKII fluorescence images. (C) Timeline of the experimental procedure. (D) Schematic diagram of chemogenetic activation of DRN‐projecting rACC CaMKII neurons. (E) Anatomical location of the rACC and a representative image showing the expression of CaMKII‐hM3D‐mCherry in the rACC. Scale bar: 200 μm. (F) Triple staining of hM3D‐mCherry (red), CaMKII (green) and DAPI (blue) in the rACC (left). Qualification of hM3D^+^/CaMKII^+^ neurons in the rACC (right) (*n* = 3). White arrows, hM3D‐mCherry, CaMKII and DAPI colabeled neurons. Scale bar: 20 μm. (G) Representative images of hM3D‐mCherry (red), c‐Fos (green) and DAPI (blue) colabeled neurons (left) and quantification of hM3D^+^/c‐Fos^+^ neurons in the rACC after CNO or saline i.p. injection (right) (*n* = 3; ***p < 0.001). White arrows, colocalization of hM3D, c‐Fos and DAPI. Scale bar: 50 μm. (H) Time rats spent in the center area (left) and total distance rats traveled (right) in the OF test (*n* = 9; **p < 0.01). (I) Time spent in the open arms in the EPM test (*n* = 9; **p < 0.01). (J) Time spent in the open arms in the EZM test (*n* = 9; *p < 0.05). (K) Timeline of the experimental procedure. (L) Schematic diagram of chemogenetic inhibition of DRN‐projecting rACC CaMKII neurons. (M) Anatomical location of rACC and representative image showing the expression of CaMKII‐hM4D‐mCherry in the rACC. Scale bar: 200 μm. (N) Triple staining of hM4D‐mCherry (red), CaMKII (green) and DAPI (blue) in the rACC (left). Qualification of hM4D^+^/CaMKII^+^ neurons in the rACC (right) (*n* = 3). White arrows, hM4D‐mCherry, CaMKII and DAPI colabeled neurons. Scale bar: 20 μm. (O) Representative images of hM4D‐mCherry (red), c‐Fos (green) and DAPI (blue) colabeled neurons (left) and quantification of hM4D^+^/c‐Fos^+^ neurons in the rACC after CNO or saline i.p. injection (right) (*n* = 3; *p < 0.05). White arrows, colocalization of hM4D, c‐Fos and DAPI. Scale bar: 50 μm. (P) Time rats spent in the open arms in the EPM test (*n* = 7; *p < 0.05, **p < 0.01). (Q) Time rats spent in the open arms in the EZM test (*n* = 8; *p < 0.05, **p < 0.01, ***p < 0.001). (R) Time rats spent in the open arms in the center area (left) and total distance rats traveled (right) in the OF test (*n* = 6–8; *p < 0.05). Data are represented as the mean ± SEM. Significance was assessed by two‐tailed unpaired Student's *t* test (G–J, O), by one‐way ANOVA with Bonferroni post hoc correction (P and R) and by Kruskal–Wallis test (Q). Aq, aqueduct.

To explore the influence of DRN‐projecting rACC neurons on chronic inflammatory pain‐induced anxiety‐like behavior, we first identified the anatomical association between the rACC and DRN. We perfused an anterograde tracer adeno‐associated virus (AAV) (AAV2/9‐CaMKII‐EGFP) into the rACC or a retrograde tracer AAV (AAV2/R‐CaMKII‐EGFP) into the DRN. The rats were sacrificed 6 weeks after the virus injection. In the anterograde tracing experiment, we observed that the fibers from the rACC CaMKII neurons (rACC^CaMKII^) were located in the DRN, and in the retrograde tracing experiment, the retrogradely transported AAV injected into the DRN spread retrogradely into the somas of rACC^CaMKII^ neurons (Figure 2A,B). Focusing on the anatomical connection between the ACC and DRN, we further confirmed the relationship between the rACC^CaMKII^‐DRN circuit and pain‐induced anxiety‐like behaviors. We manipulated the rACC^CaMKII^‐DRN circuit in behavioral assays by using a chemogenetic approach in combination with the Cre‐loxP system. To ensure full virus expression, behavioral tests were not performed until after 6 weeks had passed (Figure 2C). The Cre‐dependent anterograde AAV carrying the excited receptor hM3D linked with mCherry (AAV2/9‐CaMKII‐hM3D‐DIO‐mCherry) was delivered into the rACC, and retrograde AAV carrying Cre (AAV2/R‐CaMKII‐Cre) was injected into the DRN to specifically infect DRN‐projecting rACC CaMKII neurons (Figure 2D). We verified the injection sites, infection specificity and effectiveness of the virus (Figure 2E–G). mCherry‐labeled neurons were activated via intraperitoneal (i.p.) injection of clozapine‐N‐oxide (CNO) 30 min before each behavioral test, and rats in the control group were injected with the same volume of sterile 0.9% saline. We found that activation of DRN‐projecting rACC CaMKII neurons significantly increased rats' exploration time in the central area of the OF and the open arms of the EPM and EZM (Figure 2H–J). These results suggest that activating DRN‐projecting rACC CaMKII neurons could reverse CFA‐induced anxiety‐like behaviors.

Given the anxiolytic effect of activating the rACC^CaMKII^‐DRN circuit, we next explored whether inhibiting the rACC^CaMKII^‐DRN circuit could induce anxiety‐like behaviors. We tested the effects of inhibition of the rACC^CaMKII^‐DRN circuit in rats that underwent short‐term subthreshold pain. The same strategy as the circuit activation experiment above was applied to ensure that DRN‐projecting rACC CaMKII neurons adequately expressed the inhibitory hM4D receptor (Figure 2L). Relative to the chronic pain model rats injected with CFA 28 days before behavioral tests, rats that underwent short‐term pain reactions were injected with CFA 9 days before behavioral tests (Figure 2K). We verified the injection sites, infection specificity and effectiveness of the virus (Figure 2M–O). We found that short‐term pain induced by CFA failed to induce anxiety‐like behaviors in rats, while inhibition of DRN‐projecting rACC CaMKII neurons induced anxiety‐like behavior in rats with short‐term pain (Figure 2P–R).

### 3.3 Chemogenetic activation of the rACC^CaMKII^‐DRN^5‐HT^ circuit relieved CFA‐induced anxiety‐like behaviors in chronic pain rats

5‐HTergic and GABAergic neurons in the DRN are critical neuronal clusters involved in the regulation of negative emotion.^29^, ^30^ Anatomical studies have identified the direct monosynaptic connectivity of glutamatergic neurons from the rACC with both 5‐HTergic (DRN^5‐HT^) and GABAergic (DRN^GABA^) neurons in the DRN.^31^, ^32^ The local micronetwork within the DRN is mainly composed of 5‐HTergic neurons and GABAergic interneurons. 5‐HTergic neurons form a dynamic balance with local GABAergic neurons.^30^ Therefore, we speculated that two circuits may be related to chronic pain‐induced anxiety‐like behavior. One possibility was that the excitability of rACC^CaMKII^ neurons projecting to the DRN decreased, which in turn reduced the excitability of DRN^5‐HT^ neurons, resulting in anxiety‐like behavior. Another possibility was that the increased excitability of rACC^CaMKII^ neurons projecting to the DRN increased the excitability of DRN^GABA^ neurons, which in turn inhibited the excitability of DRN^5‐HT^ neurons, resulting in anxiety‐like behavior.

**FIGURE 3:**
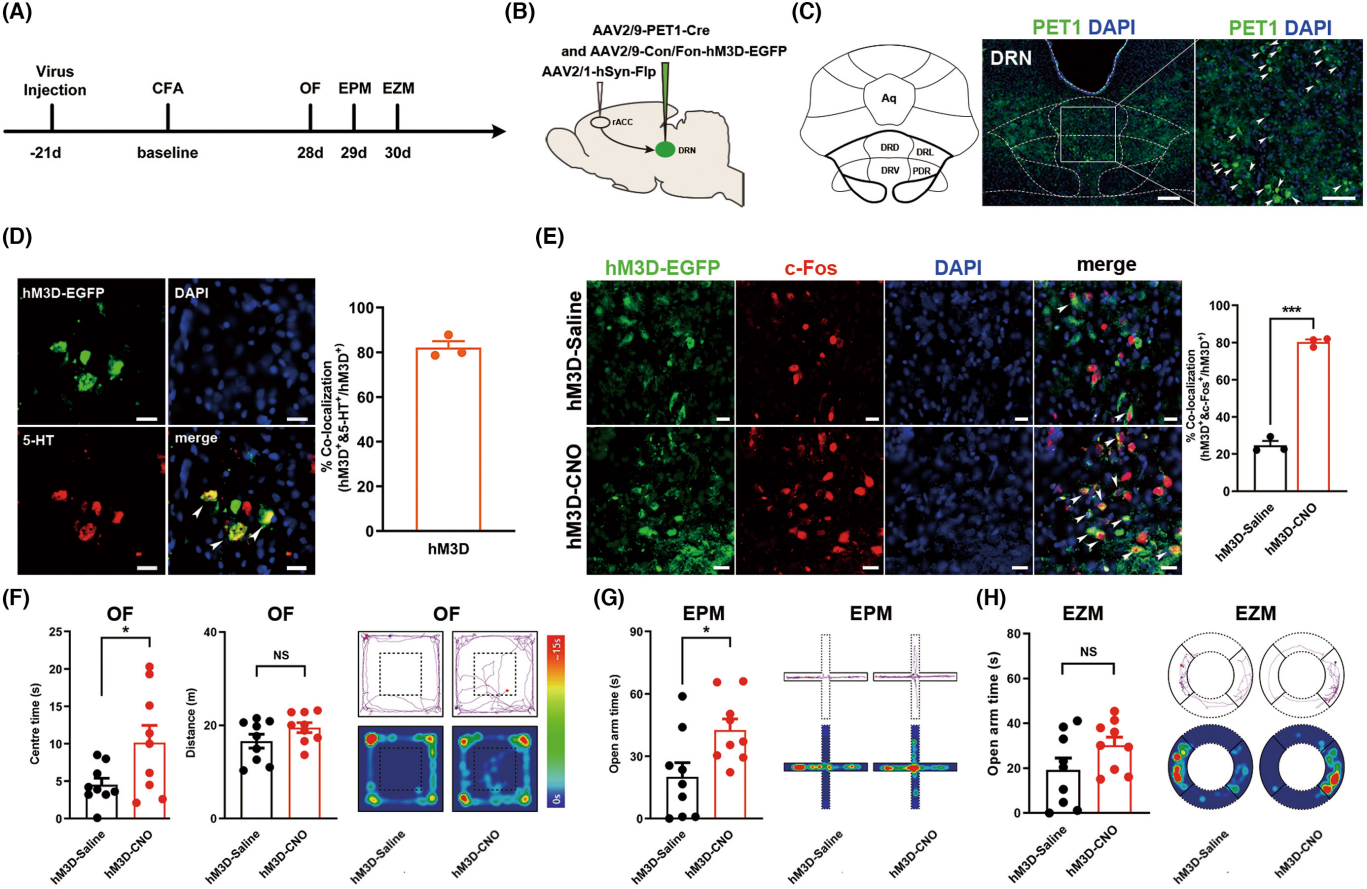
Chemogenetic activation of the rACC^CaMKII^‐DRN^5‐HT^ circuit relieves CFA‐induced anxiety‐like behaviors in the rat model of chronic pain. (A) Timeline of the experimental procedure. (B) Schematic diagram of chemogenetic activation of the rACC postsynaptic DRN 5‐HTergic neurons. (C) Anatomical location of the DRN and representative image showing the expression of Pet1‐EGFP in the DRN. Scale bars: 200 μm (left) and 100 μm (right). (D) Triple staining of hM3D‐EGFP (green), 5‐HT (red), and DAPI (blue) in the DRN (left). Qualification of hM3D^+^/5‐HT^+^ neurons in the DRN (right) (*n* = 3). White arrows, hM3D‐EGFP, 5‐HT, and DAPI colabeled neurons. Scale bar: 20 μm. (E) Representative images of hM3D‐EGFP (green), c‐Fos (red) and DAPI (blue) co‐labeled neurons (left) and quantification of hM3D^+^/c‐Fos^+^ neurons in the DRN after CNO or saline i.p. injection (right) (*n* = 3; ***p < 0.001). White arrows, colocalization of hM3D and c‐Fos. Scale bar: 20 μm. (F) Time rats spent in the central area (left), total distance rats traveled (middle), and representative trajectory map and activity heatmap (right) in the OF test (*n* = 9; *p < 0.05). (G) Time rats spent in the open arms (left) and representative trajectory map and activity heatmap (right) in the EPM test (*n* = 9; *p < 0.05). (H) Time rats spent in the open arms (left) and representative trajectory map and activity heatmap (right) in the EZM test (*n* = 9). Data are represented as the mean ± SEM. Significance was assessed by two‐tailed unpaired Student's *t* test. Aq, aqueduct; DRD, dorsal raphe nucleus, dorsal part; DRV, dorsal raphe nucleus, ventral part; DRL, dorsal raphe nucleus, lateral part; PDR, posterodorsal raphe nucleus.

We explored the role of DRN^5‐HT^ neurons receiving direct rACC inputs in chronic pain‐induced anxiety‐like behaviors. We specifically labeled DRN^5‐HT^ neurons receiving direct rACC inputs by injecting anterograde trans‐monosynaptic AAV2/1‐Flp into the rACC and a mixed solution of AAV2/9‐Pet1‐Cre (specifically labeling 5‐HTergic neurons^24^) and Cre/Flp‐dependent AAV (AAV2/9‐hSyn‐Con/Fon‐hM3D‐EGFP) into the DRN (Figure 3B). The expression of the hM3D receptor (AAV2/9‐hSyn‐Con/Fon‐hM3D‐EGFP) requires both Cre recombinase and Flp recombinase. By this strategy, the hM3D receptors could be specifically expressed on DRN^5‐HT^ neurons receiving direct rACC inputs. The model was established 3 weeks after virus injection (Figure 3A). We found that Pet1‐positive neurons receiving rACC CaMKII projections were predominantly distributed in the dorsal and ventral parts of the DRN (Figure 3C). We verified the infection specificity and effectiveness of the virus (Figure 3D,E). In the behavioral tests, rats exhibited a significant increase in exploration in the central area of the OF and the open arms of the EPM (Figure 3F,G), and there was a tendency for increased exploration in the open arm of the EZM, however, the difference was not statistically significant (Figure 3H).

Combined with the above results on activating the rACC^CaMKII^‐DRN circuit to relieve anxiety‐like behavior, we suggest that the mechanism of activation of the rACC^CaMKII^‐DRN circuit to ameliorate anxiety‐like behaviors could be further activation of postsynaptic DRN 5‐HTergic neurons.

### 3.4 Chemogenetic activation or inhibition of the rACC^CaMKII^‐DRN^GABA^ circuit failed to alter CFA‐evoked anxiety‐like behaviors in rats

**FIGURE 4:**
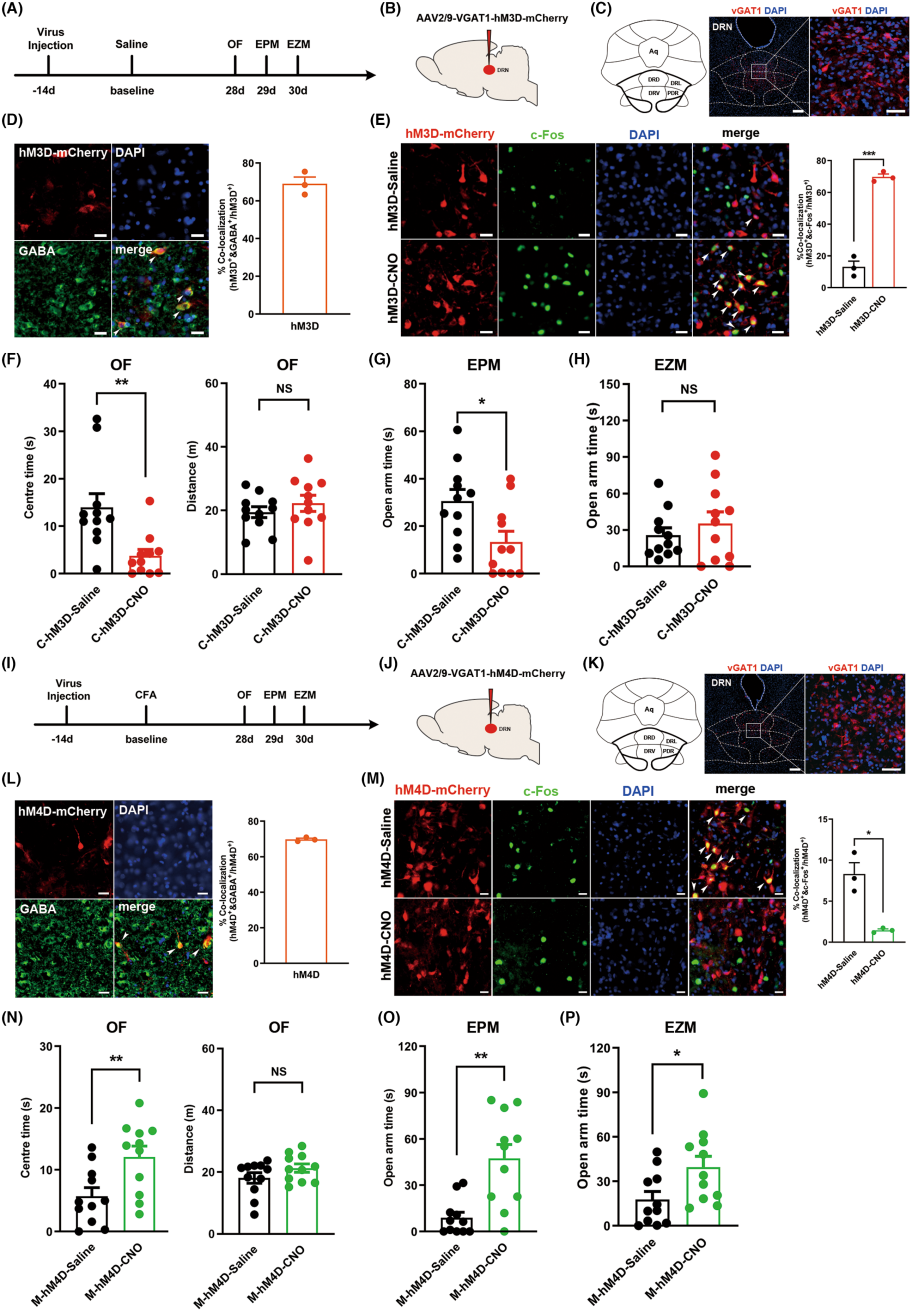
GABAergic neurons in the DRN participate in chronic pain‐induced anxiety‐like behaviors. (A) Timeline of the experimental procedures. (B) Schematic diagram of chemogenetic activation of DRN GABAergic neurons. (C) Anatomical location of the DRN and representative image showing the expression of vGAT1‐hM3D‐mCherry in the DRN. Scale bars: 200 μm (left) and 50 μm (right). (D) Triple staining of hM3D‐mCherry (red), GABA (green), and DAPI (blue) in the DRN (left). Qualification of hM3D^+^/GABA^+^ neurons in the DRN (right) (*n* = 3). White arrows, hM3D‐mCherry, GABA, and DAPI colabeled neurons. Scale bar: 20 μm. (E) Representative images of hM3D‐mCherry (red), c‐Fos (green) and DAPI (blue) colabeled neurons (left) and quantification of hM3D^+^/c‐Fos^+^ neurons in the DRN after CNO or saline i.p. injection (right) (*n* = 3; ***p < 0.001). White arrows, colocalization of hM3D, c‐Fos and DAPI. Scale bar: 20 μm. (F) Time rats spent in the center area (left) and total distance rats traveled (right) in the OF test (*n* = 11; **p < 0.01). (G) Time rats spent in the open arms in the EPM test (*n* = 11; *p < 0.05). (H) Time rats spent in the open arms in the EZM test (*n* = 11). (I) Timeline of the experimental procedures. (J) Schematic diagram of chemogenetic inhibition of DRN GABAergic neurons. (K) Anatomical location of DRN and representative image showing the expression of vGAT1‐hM4D‐mCherry in the DRN. Scale bars: 200 μm (left) and 50 μm (right). (L) Triple staining of hM4D‐mCherry (red), GABA (green), and DAPI (blue) in the DRN (left). Qualification of hM4D^+^/GABA^+^ neurons in the DRN (right) (*n* = 3). White arrows, the hM4D‐mCherry, GABA, and DAPI colabeled neurons. Scale bar: 20 μm. (M) Representative images of hM4D‐mCherry (red), c‐Fos (green), and DAPI (blue) colabeled neurons (left) and quantification of hM4D^+^/c‐Fos^+^ neurons in the DRN after CNO or saline i.p. injection (right) (*n* = 3; *p < 0.05) White arrows, colocalization of hM4D, c‐Fos and DAPI. Scale bar: 20 μm. (N) Time rats spent in the central area (left) and total distance rats traveled (right) in the OF test (*n* = 11; **p < 0.01). (O) Time rats spent in the open arms in the EPM test (*n* = 11; **p < 0.01). (P) Time rats spent in the open arms in the EZM test (*n* = 11; *p < 0.05). Data are represented as mean ± SEM. Significance was assessed by two‐tailed unpaired Student's *t* test (E, F, H, M, N and P) or by Mann–Whitney *U* test (G and O).

Based on the inhibitory effect of GABAergic neurons in the DRN on local 5‐HTergic neurons and the anatomical relationship with monosynaptic connections of rACC neurons, another possible mechanism for the anxiolytic effect of rACC‐DRN circuit activation is a reduction in the inhibitory effect on 5‐HTergic neurons due to decreased activity of GABAergic neurons, resulting in increased activity of 5‐HTergic neurons. We explored the role of DRN GABAergic neurons in chronic pain‐induced anxiety‐like behaviors. We first examined the relationship between DRN^GABA^ neurons and chronic pain‐induced anxiety‐like behaviors. Rats were injected with AAV carrying the gene encoding vesicular GABA transporter 1 (VGAT1) in conjunction with the hM3D receptor or hM4D receptor into the DRN, and behavioral studies were performed 6 weeks after virus injection (Figure 4A,B,I,J). The rats were executed 90 min after the end of behavioral tests, and immunofluorescence staining was performed to verify the specificity and efficacy of the virus infection. (Figure 4C–E,K–M). We found that chemogenetic activation of DRN^GABA^ neurons in the physiological state induced anxiety‐like behaviors in the rats (Figure 4F–H), while inhibition of DRN^GABA^ neurons alleviated chronic pain‐induced anxiety‐like behaviors (Figure 4N–P). These results suggest that DRN^GABA^ neurons are indeed involved in regulating chronic pain‐induced anxiety‐like behavior.

**FIGURE 5:**
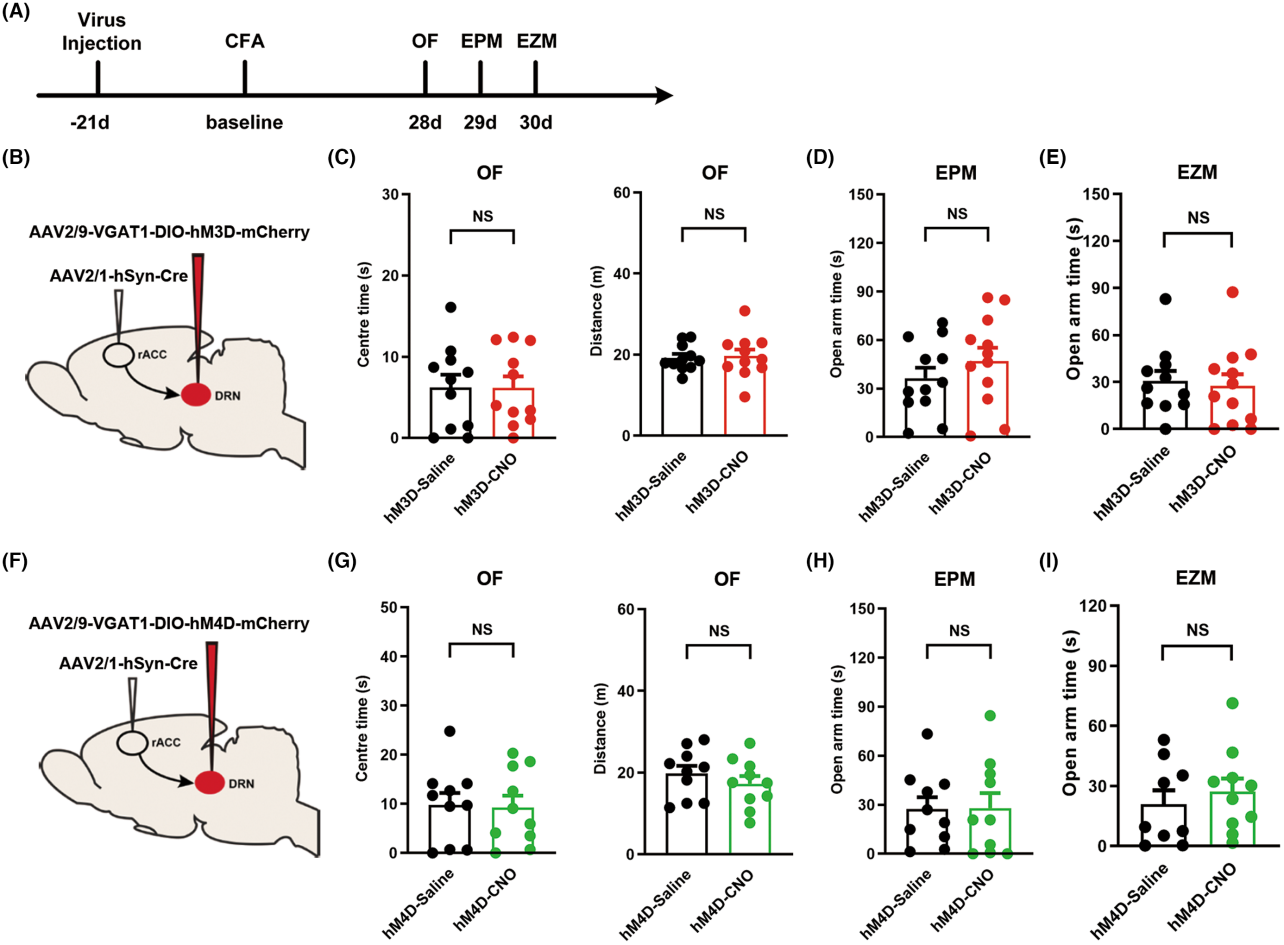
Chemogenetic activation or inhibition of the rACC^CaMKII^‐DRN^GABA^ circuit failed to alter CFA‐evoked anxiety‐like behaviors in rats. (A) Timeline of the subsequent experimental procedures. (B) Schematic diagram of chemogenetic activation of DRN^GABA^ receiving direct rACC inputs. (C) Time rats spent in the central area (left) and total distance rats traveled (right) in the OF test (*n* = 11). (D) Time rats spent in the open arms in the EPM test (*n* = 12). (E) Time rats spent in the open arms in the EZM test (*n* = 11–12). (F) Schematic diagram of chemogenetic inhibition of DRN^GABA^ receiving direct rACC inputs. (G) Time rats spent in the central area (left) and total distance traveled (right) in the OF test (*n* = 10). (H) Time rats spent in the open arms in the EPM test (*n* = 10). (I) Time rats spent in the open arms in the EZM test (*n* = 9–10). Data are represented as the mean ± SEM. Significance was assessed by two‐tailed unpaired Student's *t* test.

Next, we further explored the role of DRN^GABA^ neurons receiving rACC inputs on chronic pain‐induced anxiety‐like behaviors. Anterograde trans‐monosynaptic AAV2/1‐hSyn‐Cre was injected into the rACC, and AAV2/9‐VGAT1‐hM3D‐DIO‐mCherry was injected into the DRN, then, the rats were housed for 7 weeks to ensure adequate virus expression (Figure 5A,B). Unexpectedly, the results of behavioral tests showed that activation of DRN^GABA^ neurons receiving direct rACC inputs did not affect anxiety‐like behavior in CFA rats (Figure 5C–E). The same strategy was performed to inhibit DRN^GABA^ receiving direct rACC inputs, and no alteration was found (Figure 5F–I). These results indicate that excitatory synaptic transmission from the rACC to DRN GABAergic neurons fails to participate in the development of chronic inflammatory pain‐induced anxiety‐like behaviors.

### 3.5 EA ameliorates anxiety‐like behaviors of rats with chronic pain and reverses chronic pain‐induced changes in the activity of rACC CaMKII neurons and DRN 5‐HTergic neurons

Our previous research found that EA could relieve chronic pain‐induced anxiety‐like behaviors.^25^ Other studies have shown that EA can activate rACC^CaMKII^ and DRN^5‐HT^ neurons.^22^, ^33^, ^34^ Based on the above, we speculated that EA could intervene in chronic pain‐induced anxiety‐like behavior through the rACC^CaMKII^‐DRN^5‐HT^ circuit.

**FIGURE 6:**
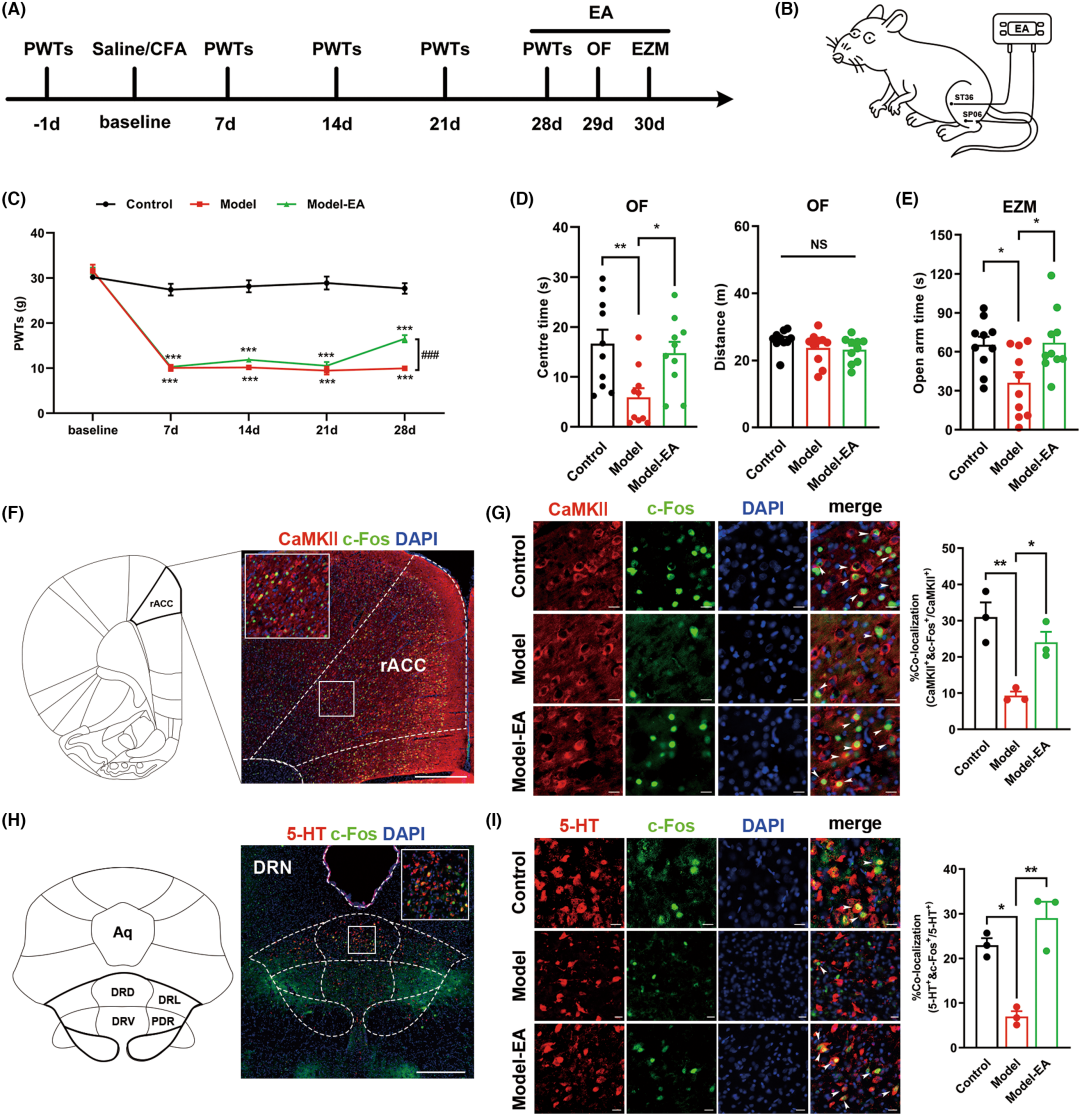
EA ameliorates anxiety‐like behaviors in the rat model of chronic pain and reverses chronic pain‐induced changes in the activity of rACC CaMKII neurons and DRN 5‐HTergic neurons. (A) Timeline of the experimental procedure. (B) Schematic diagram of EA treatment. (C) Time course of CFA‐induced changes in pain thresholds and the therapeutic effects of EA (*n* = 10; ***p < 0.001 vs. control group at the same time, ^###^ *p* < 0.001 vs. model group at 28th day). (D) Time rats spent in the central area (left) and total distance rats traveled (right) in the OF test (*n* = 10; *p < 0.05, **p < 0.01). (E) Time rats spent in the open arms in the EZM test (*n* = 10; test: *p < 0.05). (F) Anatomical location of the rACC and representative immunofluorescence staining image of CaMKII and c‐Fos positive neurons. Scale bar: 500 μm. (G) Representative images of CaMKII (red), c‐Fos (green) and DAPI (blue) colabeled neurons (left) and quantification of CaMKII^+^/c‐Fos^+^ colabeled neurons (right) in the rACC (*n* = 3; *p < 0.05, **p < 0.01). White arrows, colocalization of CaMKII, c‐Fos and DAPI. Scale bar: 20 μm. (H) Anatomical location of the DRN and representative immunofluorescence staining image of 5‐HT and c‐Fos positive neurons. Scale bar: 500 μm. (I) Representative images of 5‐HT (red), c‐Fos (green) and DAPI (blue) colabeled neurons (left) and quantification of 5‐HT^+^/c‐Fos^+^ colabeled neurons (right) in the DRN (*n* = 3; *p < 0.05, **p < 0.01). White arrows, the colocalization of 5‐HT, c‐Fos and DAPI. Scale bar: 20 μm. Data are represented as the mean ± SEM. Significance was assessed by two‐way repeated‐measures ANOVA with Bonferroni's post hoc correction between groups (C) or by one‐way ANOVA with Bonferroni post hoc correction between groups (D, E, G, I).

To evaluate the anxiolytic effects of EA treatment in rats with chronic pain, we treated model rats with EA. The bilateral Zusanli (ST36) and Sanyinjiao (SP06) acupoints were selected, and the EA intervention was administered before each behavioral test (Figure 6A,B). The results revealed that EA significantly reversed the decrease in the pain threshold induced by plantar injection of CFA (Figure 6C). For behavioral tests, EA reversed the reduced exploration in the central area of the OF and the open arms of the EZM in rats with chronic pain, with no effect on motor function (Figure 6D,E). The immunofluorescence staining was performed to observe changes in the activity of CaMKII neurons in the rACC and 5‐ HTergic neurons in the DRN. We chose c‐Fos as a marker of neuronal activation,^35^ and brain slices were subjected to fluorescent triple‐label staining. For the rACC, the results showed lower expression of CaMKII^+^/c‐Fos^+^ neurons in the model group than in the control group, while EA treatment upregulated c‐Fos expression in CaMKII^+^ neurons in the model rats (Figure 6F,G). Consistently, EA reversed the decrease in DRN 5‐HT^+^/c‐Fos^+^ neurons caused by chronic pain (Figure 6H,I). The results demonstrate that EA has anxiolytic effects and increases the activity of CaMKII neurons in the rACC and 5‐HTergic neurons in the DRN.

### 3.6 Chemogenetic inhibition of the rACC^CaMKII^‐DRN^5‐HT^ circuit reversed the therapeutic effects of EA on chronic pain‐induced anxiety‐like behaviors

Given the results above, we speculated that EA could relieve chronic pain‐induced anxiety‐like behavior by activating the rACC^CaMKII^‐DRN^5‐HT^ circuit. To further confirm the activating effect of EA on the rACC^CaMKII^‐DRN^5‐HT^ circuit, we inhibited the rACC^CaMKII^‐DRN^5‐HT^ circuit during EA to observe whether the anxiolytic effect of EA could be reversed.

**FIGURE 7:**
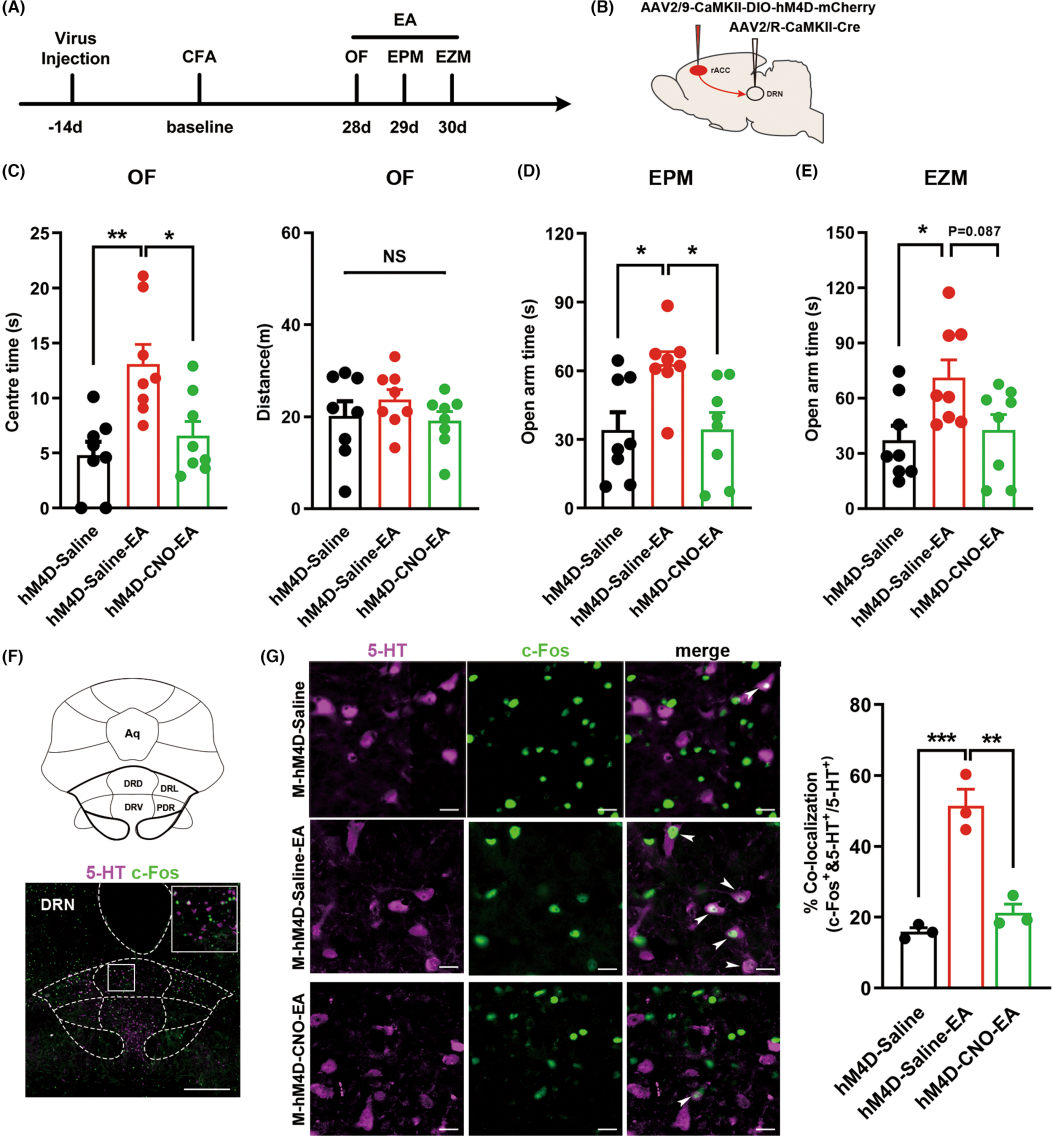
Chemogenetic inhibition of the rACC^CaMKII^‐DRN^5‐HT^ circuit reversed the therapeutic effects of EA on chronic pain‐induced anxiety‐like behaviors. (A) Timeline of the experimental procedure. (B) Schematic diagram of chemogenetic inhibition of the rACC‐DRN circuit. (C) Time rats spent in the central area (left) and total distance rats traveled (right) in the OF test (*n* = 8; *p < 0.05, **p < 0.01). (D) Time rats spent in the open arms in the EPM test (*n* = 8; *p < 0.05). (E) Time rats spent in the open arms in the EZM test (*n* = 8; *p < 0.05). (F) Anatomical location of the DRN and representative immunofluorescence staining image of 5‐HT and c‐Fos positive neurons. Scale bar: 500 μm. (G) Representative images of 5‐HT (purple) and c‐Fos (green) double‐labeled neurons (left) and quantification of 5‐HT^+^/c‐Fos^+^ neurons (right) in the DRN (*n* = 3; **p < 0.01, ***p < 0.001). White arrows, neurons colocalized with 5‐HT and c‐Fos. Scale bar: 20 μm. Data are represented as the mean ± SEM. Significance was assessed by one‐way ANOVA with Bonferroni post hoc correction between groups.

The experimental procedure is shown in Figure 7A. The Cre‐dependent AAV carrying the hM4D receptor and mCherry were perfused into the rACC, and a retrograde AAV expressing Cre was perfused into the DRN (Figure 7B). Rats were subjected to EA therapy before each behavioral test, with i.p. injection of CNO or sterile 0.9% saline administered 30 min before EA treatment. The behavioral tests showed that EA treatment increased the exploration activities of rats in the central area of the OF and the open arms of the EPM and EZM, whereas inhibition of the DRN‐projecting rACC CaMKII neurons reversed the anxiolytic effects of EA treatment (Figure 7C–E). To further verify the effect of inhibiting the rACC^CaMKII^‐DRN^5‐HT^ circuit with EA treatment, we detected changes in DRN 5‐HTergic neuronal activity. The results showed that EA treatment significantly elevated DRN 5‐HTergic neuronal activity, while inhibition of DRN‐projecting rACC CaMKII neurons antagonized EA‐induced activation of DRN^5‐HT^ neurons (Figure 7F,G). Based on these results, we conclude that activation of the rACC^CaMKII^‐DRN^5‐HT^ circuit is needed for the anxiolytic effect of EA treatment.

## 4 DISCUSSION

Positive interactions between negative emotions, such as fear, anxiety and depression, and chronic pain have been reported in both patients and animal models.^13^, ^36^, ^37^, ^38^ The ACC is involved in the modulation of both pain perception and anxiety and is thought of as one of the most important regions for the interaction between chronic pain and negative emotions.^39^ The ACC could integrate sensory inputs and emotional signaling by receiving nociceptive inputs from the spinal cord dorsal horn‐thalamus pathway and anxiety information from the subcortical regions that are involved in the regulation of emotions, such as the amygdala, lateral habenula (LHb) and DRN. Although it is generally considered that the ACC may play a key role in the comorbidity of chronic pain and anxiety, the circuit mechanisms underlying ACC‐mediated development of the comorbidity of chronic pain and anxiety disorders remain poorly understood. Specifically, little is known about the subcortical regions that are recruited by the ACC in patients with comorbid chronic pain and anxiety disorders. In this study, we revealed that the DRN was a key subcortical region that was recruited by the rACC in the development of CFA chronic pain‐induced anxiety‐like behaviors.

The serotonin system is proposed to regulate emotional states and underlies the etiology of mood disorders.^40^, ^41^, ^42^, ^43^ The DRN is the primary neuronal source of serotonin.^44^ The long‐range, top‐down control exerted by the ACC in the DRN is of particular interest because of its purported role in pain processing and mood regulation. The present study demonstrated that impairment of excitatory synaptic transmission from the rACC to the DRN was critically implicated in CFA chronic pain‐induced anxiety‐like behaviors. Chemogenetic activation of the rACC‐DRN circuit alleviated CFA‐induced anxiety‐like behaviors, whereas chemogenetic inhibition of the rACC‐DRN circuit resulted in short‐term CFA pain reaction induction of anxiety‐like behaviors. Furthermore, we revealed that the development of CFA chronic pain‐induced anxiety‐like behaviors is attributed to the dysfunction of rACC CaMKII neurons projecting to DRN serotonergic neurons (rACC^CaMKII^‐DRN^5‐HT^) but not rACC CaMKII neurons projecting to DRN GABAergic neurons (rACC^CaMKII^‐DRN^GABA^).

The local micronetwork within the DRN is composed of both glutamatergic pyramidal neurons and GABAergic interneurons. Electrophysiological and anatomical tracing have demonstrated that both DRN^5‐HT^ and DRN^GABA^ neurons receive glutamatergic monosynaptic inputs from the rACC and that GABAergic neurons can modulate the function of DRN serotonergic neurons by feedforward inhibition (FFI).^31^, ^32^, ^45^, ^46^ Surprisingly, we found that excitatory synaptic transmission from the rACC to DRN GABAergic neurons failed to participate in the development of CFA chronic pain‐induced anxiety‐like behaviors because neither chemogenetic activation nor chemogenetic inhibition of rACC^CaMKII^‐DRN^GABA^ circuit had a significant effect on CFA chronic pain‐induced anxiety‐like behaviors. Several possibilities may account for this. First, the failure of rACC^CaMKII^‐DRN^GABA^ neurons in the regulation of CFA chronic pain‐induced anxiety‐like behaviors may be due to fewer projections of rACC^CaMKII^ neurons to DRN^GABA^ neurons. Indeed, it has been reported that rACC glutamatergic neurons have higher connectivity rates and provide stronger excitatory inputs to DRN^5‐HT^ neurons compared to DRN^GABA^ neurons.^31^, ^46^, ^47^ Second, the impairment of excitatory synaptic transmission from rACC to DRN^GABA^ neurons induced by CFA‐induced chronic pain may be counteracted by either decreasing inhibitory synaptic transmission to DRN^GABA^ neurons from the lateral hypothalamus^31^ or increasing excitatory transmission to DRN^GABA^ neurons from the LHb^48^ following CFA‐induced chronic pain. Last, decreased excitatory synaptic transmission from rACC^Glu^ neurons to DRN^GABA^ neurons induced by CFA‐induced chronic pain may also be reversed by decreasing cannabinoid receptor 1 (CB1R) receptor signaling in the rACC‐DRN circuit. Glutamate synapses from the mPFC onto the DRN have been shown to be modulated by endocannabinoids acting on presynaptic CB1Rs.^32^, ^49^ CB1R activation displays a stronger inhibitory effect on mPFC synapses onto GABAergic neurons than mPFC synapses onto serotonergic neurons.^32^ This differential target sensitivity allows CB1R activation to favor a net increase in serotonergic neuron output at the expense of FFI.^32^ Thus, a decrease in CB1R signaling in the rACC‐DRN circuit could reverse CFA‐induced chronic pain‐evoked attenuated excitatory transmission from rACC neurons to DRN GABAergic neurons. A decrease in CB1R signaling has been shown to cause mood disorders, such as depression.^50^, ^51^, ^52^, ^53^ Further studies are needed to elucidate how excitatory synaptic transmission from rACC neurons to DRN GABAergic neurons fails to participate in the development of CFA chronic pain‐induced anxiety‐like behaviors.

Subpopulations of DRN 5‐HTergic neurons, defined by anatomical location, function differently.^54^ In this study, Pet1‐labeled 5‐HTergic neurons were predominantly distributed in the ventral and dorsal parts of the DRN, and activation of those neurons receiving rACC projections alleviated anxiety‐like behaviors. It has also been reported that Sert‐labeled 5‐HTergic neurons were predominantly distributed in the lateral wings of the DRN and that optogenetic activation of these 5‐HTergic neurons in the DRN induced anxiety‐like behaviors.^55^ We hypothesize that this different functional role above may be related to the different spatial distribution of 5‐HTergic neurons in the DRN.

EA is increasingly used to treat chronic pain and emotional disorders that are processed in higher brain structures, including cortical areas.^2^, ^13^, ^56^ How peripheral electrical acupoint stimulations can exert an effect on such complicated diseases involving cortical and subcortical regions is poorly understood. Although the molecular and circuit mechanisms underlying peripheral electrical acupoint stimulation induction of alterations in the activity of the central nervous system are still unclear, substantial evidence suggests that peripheral electrical acupoint stimulations activate cortical and subcortical regions when activity is measured by the c‐Fos expression test^57^, ^58^ and functional magnetic resonance (fMR) imaging.^59^, ^60^, ^61^, ^62^ Additionally, there is evidence that EA exerts its antistress and psychosomatic effects through alterations in the function of the dopaminergic and serotonergic systems in the cortex and DRN.^22^, ^63^ Our previous study also found that EA alleviates inflammatory pain‐induced conditioned place aversion behavior by regulating rACC synchronous neural oscillations.^21^ In the present study, we demonstrated that EA ameliorates anxiety‐like behaviors in rats with chronic pain by restoring the impaired function of rACC CaMKII neurons projecting to DRN serotonergic neurons.

Theoretically, there are at least two possible mechanisms by which EA might regulate the activity of the rACC^CaMKII^‐DRN^5‐HT^ circuit: presynaptic alteration of glutamate release and postsynaptic change in synaptic strength. Presynaptic alteration of glutamate release might underlie the effect of EA on chronic pain‐induced anxiety^2^ because pharmacological alteration of presynaptic mechanism‐induced LTP displays anxiolytic and analgesic effects in chronic pain models,^64^ while pharmacological change in postsynaptic mechanism‐induced LTP has only analgesic effects.^65^ A potential mechanism by which EA may exert an effect on presynaptic glutamate release is G protein‐coupled receptor (GPCR) function regulation. Presynaptic GPCRs robustly regulate information flow in neural circuits by modulating presynaptic transmitter release probability.^53^, ^66^, ^67^ Several GPCRs, such as κ‐opioid receptors and CB1Rs, have been shown to be involved in emotion disorders by presynaptically suppressing glutamate release.^53^, ^68^ Changes in the activity of these GPCRs and their endogenous ligand expression may be a potential mechanism by which chronic pain yields anxiety disorders. EA has been shown to modulate the expression of dynorphine, an endogenous ligand of KOR.^69^ Further studies are needed to elucidate the molecular and signaling mechanisms by which EA activates the rACC^CaMKII^‐DRN^5‐HT^ circuit.

In summary, this study has identified a novel neuronal circuit associated with anxiety‐like behaviors induced by chronic inflammatory pain and first demonstrated that the anxiolytic effect of EA on rats with chronic inflammatory pain depends on the activation of rACC CaMKII neurons projecting to DRN serotonergic neurons. Our findings may lead to the development of pharmacological strategies for the treatment of anxiety disorders associated with inflammatory pain and provide a neurobiological basis for EA treatment of such mood disorders.

## AUTHOR CONTRIBUTIONS

J.‐Q.F conceived the project and provided funding acquisition. J.‐G.L. and X.‐M.S. designed and supervised the experiments. Z.‐M.W. performed the experiments with the help of Z.S., Y.‐L.X., S.‐Q.X., J.‐Y.Y., H.‐Y.Z., X.‐Y.M., Y.‐C.Z., and X.‐X.Z. Z.‐M.W, Z.S., and S.‐Z.C. performed the data analysis. Y.‐L.J. and J.‐F.F. visualized the data. B.‐Y.L. and X.‐F.H. administrated the project. Z.‐M.W, Z.S., and S.‐Z.G. wrote the original draft. J.‐G.L. and X.‐M.S. reviewed and edited the manuscript.

## FUNDING INFORMATION

This work was supported by grants from the National Natural Science Foundation of China (82274635, 82074518 and 82074541) and the Zhejiang Provincial Natural Science Foundation of China (LY21H270010).

## CONFLICT OF INTEREST STATEMENT

The authors declare no competing interests.

## Data Availability

The data that support the findings of this study are available from the corresponding author upon reasonable request.
